# Texas State Medical Association

**Published:** 1899-03

**Authors:** 


### Texas State Medical Association.

Office of the President, Terrell, Texas, Feb. 28, 1899.

Dear Doctor:—The Texas State Medical Association will convene in San Antonio on the 25th (fourth Tuesday) of April.

The meeting promises to be of unusual interest.

It is confidently expected that all the Sections will be full, the papers and discussions of great practical value. The benefit derived from section work cannot be overestimated. We obtain in a brief, pointed and practical manner the cream of the conclusions derived from the careful and painstaking observations of the best talent of the State.

It is quite probable that many subjects of moment to the profession will come up for consideration.

This should be the representative medical body of Texas. Every regular, ethical physician within its borders should be a member. It is a duty he owes to himself, to his State and his profession. It is of utmost importance that the medical profession of Texas be thoroughly organized. It can have no standing or influence in public matters concerning medical subjects without such organization. Every self-respecting physician should feel a pride in helping to keep his own State in line with any other State in medical progress.

The profession of Texas should unite in trying to make its own State organization the best in the land, that its influence be felt for good in every department of government, that it be instrumental in shaping such laws as pertain to medicine and the allied sciences which will promote the best interests of the public welfare.

Then come with us to San Antonio in April, that beautiful month of delightful breezes and fragrant flowers, when dancing sunbeams, tempered by the cooling zephyrs from the great gulf, reflect in sublime grandeur the glory of the great Creator.

Come view the spot where Ben Milam, Travis, Bowie and Crockett, with their little band of matchless heroes, sacrificed their lives on the altar of Texas independence and shed imperishable renown upon Texas valor. The Alamo with its bloody history still stands a monument to more than Spartan heroism. In this historic old city let the profession of Texas make its pilgrimage in April next, and kneel with reverence at the shrine of Scientific Medicine.

J. T. Wilson, M. D.,
President, T. S. M. A.

---

Galveston, Texas, February 24, 1899.

Dear Doctor:—We desire to remind you that the time (April 25th) is rapidly approaching for the next meeting of the State Medical Association; and to ask you to prepare a paper for the Section on General Medicine. If it will be possible for you to do so, please send us the title by March 15th, so that it can be placed upon the program.

Respectfully yours,
W. F. Starley, Secretary.
Geo. H. Lee, Chairman.

---

Austin, Texas, March 14, 1899.

Dear Doctor:—We would be glad to have you contribute a paper to the Section on Gynecology at the coming meeting of the Texas State Medical Association in San Antonio, April 25th, 26th, 27th and 28th. This section, as you know, is one of the most important from a practical standpoint, and is worthy of your careful consideration. We are very anxious to have it well represented at the coming meeting, and we trust that we can depend on your assistance. Please send us title of your paper at an early date, very greatly obliging,

Yours fraternally,

A. N. Denton, Chairman of Section,

Austin, Texas.

S. E. Hudson, Secretary of Section,

Austin, Texas.

BELTON, TEXAS, February 21, 1899.

DEAR DOCTOR:—We wish to remind you of the approaching meeting of the State Medical Association at San Antonio on April 25th, 26th and 27th, and at the same time to solicit from you a paper for the Section on Obstetrics and Diseases of Children.

Please send your paper, or its title, to the Secretary two weeks before the meeting, so that it may have its proper place in the published program.

Help us to give this section the prominence which its importance in general practice deserves.

Fraternally and affectionately,

C. M. ALEXANDER, Chairman, Coleman.

J. M. FRAZIER, Secretary, Belton.

